# How Did the Dietary Behavior of Older Korean Adults Change During the COVID-19 Pandemic?

**DOI:** 10.3390/nu17121973

**Published:** 2025-06-11

**Authors:** Yong-Seok Kwon, Dasol Kim, Hee-Sook Lim

**Affiliations:** 1 Department of Food Sciences, National Institute of Crop and Food Science, Rural Development Administration, Wanju 55365, Republic of Korea; selenium2012@korea.kr (Y.-S.K.); rlekthwl2729@korea.kr (D.K.); 2 Department of Gerontology, AgeTech-Service Convergence Major, Graduate School of East-West Medical Science, Kyung Hee University, Yongin 17104, Republic of Korea

**Keywords:** COVID-19, Korean older adults, KNHANES, dietary behavior

## Abstract

Objectives: This study comparatively evaluated changes in the food habits and dietary patterns of adults aged ≥65 years before and during the coronavirus disease 2019 (COVID-19) pandemic using a retrospective study. Methods: Data covering the 2018–2021 period were derived from the Korea National Health and Nutrition Examination Survey. Results: Comparing the 2943 individuals in the “Before-COVID-19” individuals with the 2916 people in the “During COVID-19” group, the frequency of drinking four or more times a week decreased among the older adults during the pan-demic, as did the frequency of eating three meals a day and eating-out at least once a week. On the other hand, food security improved during the pandemic compared with before the pandemic. During the pandemic, the intake of cereals and grains decreased, while that of potatoes and starches, legumes, vegetables, eggs, milk and dairy products, and oils and fats increased. Although energy and carbohydrate intake decreased, protein, fiber, calcium, phosphorus, magnesium, potassium, zinc, riboflavin, vitamin E, vitamin C, folic acid, and fat intake increased. However, the intake of vitamin A, vitamin C, and calcium remained lower than the estimated adequate requirement of the Dietary Reference Intakes for Koreans. Additionally, the rate of nutritional insufficiency “During COVID-19” (20.76%) was 1.31–1.42 times higher than that “Before COVID-19” (16.45%). Even in models that adjusted for other factors, the rate of nutritional insufficiency was higher during the pandemic than before. Conclusions: Based on these findings, measures such as dietary education programs and guidelines for proper nutrient intake should be formulated to prevent imbalances in nutrient intake among older Koreans.

## 1. Introduction

Coronavirus disease 2019 (COVID-19), an infectious disease caused by severe acute respiratory syndrome coronavirus 2, was first identified in December 2019 in Wuhan, China, and has since spread worldwide [1]. In January 2020, the World Health Organization (WHO) declared it an international public health emergency. Subsequently, on 11 March 2020, COVID-19 was elevated to pandemic status [1,2,3]. To contain the spread of this contagious respiratory disease, numerous countries implemented diverse measures, including social distancing, movement restrictions, diagnostic testing, quarantine, and vaccination [2,4,5,6].

According to several studies, the level of physical activity decreased owing to the COVID-19 pandemic, mainly because of lockdown measures and social distancing [7,8,9,10]. In Poland, lockdown measures implemented to restrict movement owing to the COVID-19 pandemic resulted in significant increases in weight gain, meal frequency, and snack consumption [11]. Another study conducted abroad revealed that during the COVID-19 pandemic, restrictions on daily activities led to people spending more time at home instead of participating in their usual activities. Changes in lifestyle habits, including weight gain, reduced physical activity, and an increase in home-cooked and delivered meals, were also reported [12]. Meanwhile, the COVID-19 pandemic also affected food security globally. Household food insecurity reportedly increased in 2020, a period when the pandemic persisted in certain countries, including the United Kingdom and United States [13,14,15]. Such economic vulnerability and the collapse of food distribution networks owing to pandemic conditions, such as movement restrictions and lockdowns, are presumably related to various dietary problems, as reported by several previous studies [15,16,17].

On the other hand, South Korea’s COVID-19 prevention efforts received considerable international attention in the early days of the pandemic (February and March 2020) owing to South Korea’s geographical proximity to China and the frequent person-to-person exchanges, including travel and trade [18,19]. In the second half of 2020, South Korea reported fewer COVID-19 cases and deaths than other industrialized countries. Although social gatherings were restricted owing to social distancing measures, South Korea did not impose a strict national lockdown at that time. However, as in other countries, COVID-19 affected South Koreans’ daily lives, including their diets [2,20,21]. Studies comparing changes in health behaviors and dietary habits among Koreans before and during the COVID-19 pandemic have been reported. In a previous study using data from the Korea Youth Health Behavior Online Survey (2019–2020), adolescents aged 12–18 years increased the frequency of breakfast consumption and strength training during the COVID-19 pandemic while decreasing the frequency of aerobic exercise and consumption of fruits, fast food, and carbonated and sweet beverages [22]. In adults, weight and waist circumference increased during the COVID-19 pandemic, while physical activity, the frequency of breakfast consumption, the frequency of eating out, and total energy intake decreased [3,23,24]. In the case of the older adults, it has been reported that the frequency of going out decreased during the COVID-19 pandemic due to social distancing, self-quarantine, and the closure of welfare facilities for the elderly and that psychological depression increased as a result of living at home [25]. Other previous studies found that average sleep duration increased and the frequency of eating out decreased, but dietary habits were little affected [26,27].

In this study, we hypothesized that social distancing, reduced outdoor activities, and the fear of disease owing to the COVID-19 pandemic might have affected the dietary habits of older Koreans, a vulnerable group. Previous studies on COVID-19 and the dietary habits of older Koreans have primarily focused on nutrient intake during the pre-pandemic (2019) and pandemic (2020) periods [26,28]. Therefore, previous studies are considered to have limitations in inferring the pandemic’s impact on the dietary habits of older Koreans. This is because these studies all compared data from 2019 and 2020 and are, therefore, limited in identifying the long-term impact of the COVID-19 pandemic [26,28]. For this reason, this study compared the dietary habits, energy and macronutrient intakes, and food group intakes of older Koreans between two time periods: “Before the COVID-19 pandemic (2018–2019)” and “During the COVID-19 pandemic (2020–2021)” using data from the Korea National Health and Nutrition Examination Survey (KNHANES). Ultimately, we endeavored to provide baseline data for establishing effective diet and nutrition policies to prepare for similar health crises in the future.

## 2. Materials and Methods

### 2.1. Study Population and Design

This cross-sectional study used 2018–2021 KNHANES data. The KNHANES is a continuous surveillance system that assesses the nutritional health status of and prevalence of chronic diseases in a vast, representative sample of the Korean population. The KNHANES, conducted annually by the Korea Disease Control and Prevention Agency, comprises a health interview, health examination, and nutrition survey [29,30].

**Figure 1:**
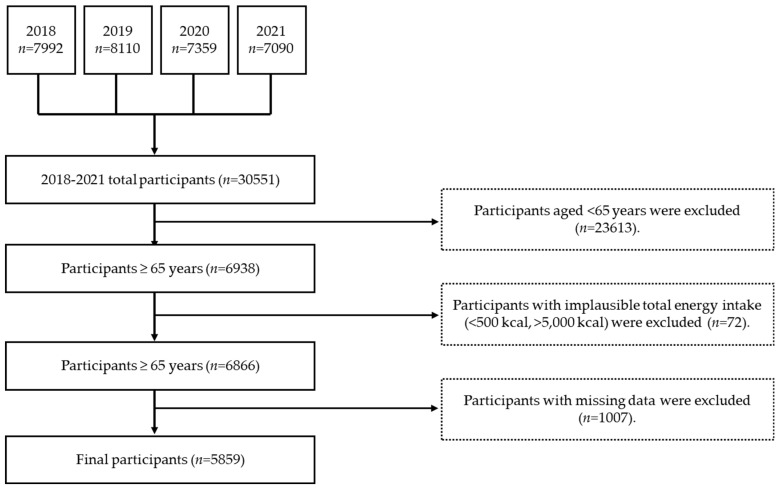
Participant selection flowchart.

Research participants were selected based on previous studies [30,31,32], and the process was as follows: The study used raw data (*n* = 30,551) from the 2018–2021 KNHANES. The main study population comprised older adults (age: ≥65 years) who had participated in the health and dietary surveys (24 h recall survey; *n* = 6938). Based on methods used in previous studies [31,32], participants with a total daily calorie intake <500 or >5000 Kcal (*n* = 72; i.e., participants with an insufficient or unusually high dietary intake) were excluded from this study. In addition, dietary survey participants with missing data (*n* = 1007) were also excluded from the analysis. Ultimately, 5859 participants were selected (Figure 1). This study was approved by the Institutional Review Board (IRB) of the Korea Disease Control and Prevention Agency <IRB approval numbers: 2018 KNHANES, 2018-01-03-P-A (approval date: 12 January 2018.); 2019 KNHANES, 2018-01-03-C-A (approval date: 19 December 2018.); 2020 KNHANES, 2018-01-03-2C-A (approval date: 26 June 2020.); 2021 KNHANES, 2018-01-03-5C-A (Approval date: 23 April 2021.)>.

This study’s independent variable (i.e., the pre-pandemic-pandemic period) was classified as follows: “Before COVID-19” (2018–2019) and “During COVID-19” (2020–2021) approval date: 3 January 2018.

### 2.2. General Characteristics

This analysis was conducted based on the respondents’ gender, age, marital status, education level, region, employment status, and household income. Age categorization was based on the following research. Research on food security and related characteristics reveals that South Korea’s rapidly aging population has led to an expansion of the older population’s age range. The accelerated growth of the over-65 population, especially those aged ≥75 years, has rendered it difficult to consider those aged ≥75 years part of a homogeneous group encompassing those aged 65–74 years [33]. In addition, according to the report of 2017 Investigation of the Older Adults Condition, the proportion of the over-65 population aged 65–74 years is approximately 57%, while that of the over-65 population aged >75 years approximates 43% [33,34]. Therefore, this study categorized the participants into 65–74 and >75-year age groups. Marital status was classified as single or married; education level as high school graduate or lower, community college graduate, or four-year college graduate or higher; and occupational status as employed or unemployed. Region was categorized into urban and rural (based on town, city, or country) and household income level into upper, upper-middle, middle-lower, and lower.

### 2.3. Health Behavior

Health behavior data were collected on five key indicators: smoking status, alcohol consumption, stress level, exercise frequency, and weight status. Smoking status was classified as “never smoker”, “former smoker”, or “current smoker”. Drinking frequency was classified into the following four categories based on the 1-year drinking frequency questionnaire included in the KNHANES: “four or more times per week”, “2–3 times per week”, “1–4 times per month”, and “less than once per month”. Based on the “Usually Perceived Stress Level” questionnaire included in the KNHANES, stress level was also classified into four categories: “severe stress”, “moderate stress”, “mild stress”, and “no stress”. Exercise frequency was classified into four categories: “less than 1 day per week”, “1–2 days per week”, “3–4 days per week”, and “more than 5 days per week”. Weight status was classified according to the standards of the WHO’s Asia–Pacific Region and Korean Society for the Study of Obesity [35,36]. Body mass index (BMI) values of <18.5, 18.5–23.0, 23.0–25.0, and >25.0 kg/m^2^ were classified as underweight, normal, overweight, and obese, respectively.

### 2.4. Dietary Habits

Dietary habits were surveyed using five questions, covering breakfast frequency, snack consumption, cooking location, frequency of eating out, and food security. Food preparation location was classified using the “n_mtype” variable, which considered whether or not the food was eaten, aligning with previous studies’ categorizations [32]. “Home meals” refer to food and packed lunches prepared at home, whereas “Commercial location” refers to meals prepared at restaurants, general purchased foods, and convenience foods. Meals and free meals prepared by schools, industries, and senior centers, among others, were classified as “Institution”. Breakfast frequency was categorized into “5–7 times per week”, “3–4 times per week”, “1–2 times per week”, and “rarely”. The response options for snack consumption were “yes” and “no”. Meal provision locations were classified into home, commercial, and institutional locations. The frequency of eating out was divided into six categories: “more than once a day”, “5–6 times a week”, “3–4 times a week”, “1–2 times a week”, “1–3 times a month”, and “rarely”. Food insecurity has been included in the dietary questionnaire since the 2005 KNHANES. Responses to the question “Which of the following best describes your family’s diet in the past year?” were classified into the “Sufficiently food secure” category (based on the description, “everyone in our family could eat enough of a variety of foods—as much as they wanted”) and “Mildly food insecure” category (based on the description “everyone in our family could eat enough food but of limited variety”), according to previous studies [37,38]. The “Moderately/severely insecure” category was based on the description “food was sometimes or often insufficient because of economic challenges”.

### 2.5. Food and Nutrient Intake

In this study, 24 h dietary recall data were collected by trained dietitians in the participants’ homes as part of the KNHANES dietary survey. Participants were asked to provide detailed information on all foods and beverages consumed during the previous 24 h via a standardized and structured interview. During the interview, trained dietitians used food models, photographs, and other visual aids to help participants recall the types and amounts of foods and beverages they had consumed [31].

Food intake was categorized into 17 food groups: cereals and grain products, potatoes and starch, sugar and confectionery, legumes and legume products, seeds and nuts, vegetables, mushroom, fruits, meat, poultry and poultry products, eggs, fish and shellfish, seaweed, milk and dairy products, fats and oils, beverages, seasonings, and other foods. The intake of each food group and each participant’s total food intake were calculated. Additionally, the total intake of fresh fruit (excluding jams, compotes, and fruit juices) and that of non-starchy vegetables (excluding salted vegetables and vegetable juices) were calculated [39]. The intake of fresh fruits and non-starchy vegetables was calculated in comparison with the intake standards recommended by the WHO and World Cancer Research Fund (WCRF) [40,41].

The dietary data were linked to the Food Composition Table (9.1 revised edition) established by the National Institute of Agricultural Sciences, Rural Development Administration in Korea [42], to calculate the energy and nutrient intakes (carbohydrate, protein, fat, fiber, calcium, phosphorus, sodium, potassium, zinc, magnesium, iron, vitamin A, carotene, retinol, thiamine, riboflavin, niacin, folic acid, vitamin D, vitamin E, and vitamin C) of each participant.

Nutritional insufficiency criteria followed the Korea Disease Control and Prevention Agency’s definition, as published in the National Health Statistics [43,44]. Participants whose energy intake was <75% of the estimated energy requirement (EER) and those whose intake of iron, calcium, vitamin A, and vitamin B_2_ was less than the estimated average requirement (EAR) were classified as nutritionally insufficient.

### 2.6. Statistical Analysis

KNHANES data were collected using stratified multistage sampling rather than simple random sampling. Briefly, to ensure accurate statistical analysis, the collected data were weighted and adjusted for stratification (using KSTRATA) and clustering (using primary sampling units), according to the statistical analysis procedure outlined in the KNHANES guidelines [45]. All analyses were performed using the Statistical Analysis System survey procedure (SAS, ver. 9.4; SAS Institute, Cary, NC, USA). The participants’ general characteristics, health-related behaviors, and dietary behaviors were subjected to frequency analysis (using the SURVEYFREQ procedure). The results are expressed as frequencies and percentages (weighted percentages). The food and nutrient intake analysis results are presented as the mean ± standard error (SE) via descriptive analysis (using the SURVEYMEANS procedure). Sex, age, and energy intake were used as adjustment variables. The relationship between pre-pandemic and during-pandemic nutritional insufficiency was assessed using logistic regression analysis (via the SURVEYLOGISTIC procedure), with results presented as odds ratios (ORs) and 95% confidence intervals (CIs). Additionally, the influences of sex, age, drinking status, stress level, weight status, smoking status, food security, snack consumption, eating-out frequency, breakfast frequency, education level, household income, and marital status were adjusted in stages prior to multiple logistic regression analysis.

## 3. Results

### 3.1. General Characteristics

Table 1 shows the general characteristics of the survey participants. No significant difference in sex, age, marital status, education level, region, or household income was noted between the “Before COVID-19” and “During COVID-19” groups. However, employment status significantly differed between the two groups (*p* = 0.003), demonstrating a 5.66% increase from “Before COVID-19” to “During COVID-19”.

### 3.2. Health-Related Behaviors Before vs. During COVID-19

Pre- and during-pandemic health-related behaviors are shown in Table 2. Smoking status, stress level, exercise, obesity, or chewing ability did not significantly differ between the “Before COVID-19” and “During COVID-19” groups. However, there was a significant difference in the frequency of drinking between the two periods (*p* = 0.006). The proportion of participants who were drinking four or more times a week decreased by 1.92%, while the proportion of participants who were not drinking even once a month increased by 4.78%.

### 3.3. Dietary Behaviors Before vs. During COVID-19

Pre- and during-pandemic dietary behaviors are shown in Table 3. No statistically significant difference in snack or home-meal consumption occurred. Breakfast consumption frequency significantly decreased from “Before COVID-19” to “During COVID-19” among those consuming breakfast more than three times a week (*p* < 0.001); conversely, the frequency of eating breakfast less than two times a week increased. Regarding daily meal patterns, no significant differences were observed; however, the rate of the three-meal-a-day pattern (“B + L + D”) decreased by approximately 2.49% from “Before COVID-19” to “During COVID-19”, whereas that of two-meal-a-day patterns (“B + L”, “B + D”, and “L + D”) increased. In addition, the percentage of older adults using food service locations (where meals are prepared) is as follows. The frequency of eating food served in institutional (*p* < 0.001) and commercial (*p* = 0.0715) locations decreased by 3.26%p and 3.73%p, respectively, when comparing the “Before COVID-19” period to the “During COVID-19” period. The frequency of eating food provided at home (*p* = 0.6488) slightly increased by about 0.28 percentage points when comparing “Before COVID-19 (95.82%)” and “During COVID-19 (96.10%)”. In terms of the frequency of eating out, a significant difference was noted between the “Before COVID-19” and “During COVID-19” groups (*p* < 0.001). Specifically, “Once a day”, “5–6 times a week”, “3–4 times a week”, and “1–2 times a week” all exhibited a decrease “During COVID-19” compared with that “Before COVID-19”, whereas “1–3 times a month” and “Rarely” displayed an increase. Finally, food security increased between the pre- and during-pandemic periods; in contrast, the number of participants reporting food insecurity decreased (*p* = 0.006).

### 3.4. Food Group Intake Before vs. During COVID-19

Table 4 presents the pre- and during-pandemic daily food group intakes. The following food groups “Total Food”, “Potatoes and starches”, “Legumes”, “Vegetables”, “Eggs”, “Milks and dairy products”, and “Oils and fats” were more common “During COVID-19” than “Before COVID-19”. Generally, food group intake significantly increased (unadjusted *p*-value < 0.05 and adjusted *p*-value < 0.05; however, only the adjusted *p*-value for “Total Food” intake was significant). Conversely, “Cereals and grains” intake significantly decreased by approximately 18.87 g “During COVID-19” compared with that “Before COVID-19” (adjusted *p*-value < 0.01).

### 3.5. Plant Food Intake Before vs. During COVID-19

Table 5 presents the analysis of plant food intake (i.e., fresh fruits and non-starchy vegetables). The KNHANES’ fruit category includes candied fruits and fruit juice, while its vegetable category comprises kimchi, salted vegetables, and vegetable juice. In this study, data excluding candied fruits, fruit juice, and salted vegetables were subjected to analysis. Although fresh fruit intake was not significantly different between the two time periods, it was 8.13 g lower “During COVID-19” (157.33 g) than that “Before COVID-19” (165.46 g). Moreover, non-starchy vegetable intake was lower than that “Before COVID-19” (184.91 g). On analyzing the period “During COVID-19” (199.37 g), intake significantly increased by approximately 14.48 g (unadjusted *p*-value = 0.0233 and adjusted *p*-value = 0.004). No significant difference in the combined intake of fresh fruit and non-starchy vegetables was noted; nonetheless, it increased by approximately 6.33 g “During COVID-19” (356.70 g) compared with that “Before COVID-19” (350.37 g).

### 3.6. Nutrient Intake Before vs. During COVID-19

Pre- and during-pandemic nutrient intake results are shown in Table 6. Compared with that “Before COVID-19”, the intake of energy, carbohydrates, iron, and thiamine significantly decreased “During COVID-19” (unadjusted *p*-value < 0.001 and adjusted *p*-value < 0.001). In contrast, the energy contribution of protein, fat, fiber, calcium, phosphorus, magnesium, potassium, zinc, riboflavin, vitamin E, vitamin C, and folic acid exhibited a significantly increasing trend (unadjusted *p*-value < 0.05 and adjusted *p*-value < 0.05).

### 3.7. Effects of the Pre- and During-COVID-19 Pandemic Periods on Nutritional Insufficiency

Table 7 shows the associations of the pre- and during-pandemic periods with nutritional insufficiency. Models 1–4 revealed relationships between the pre- and during-pandemic periods and undernutrition. The nutritional insufficiency rate “During COVID-19” (20.76%) was 1.31–1.42 times higher than that “Before COVID-19” (16.45%). In particular, an independent and significant relationship existed between the pre- and during-pandemic periods and nutritional insufficiency after adjusting for sex, age, smoking, drinking status, stress, weight status, food security, snack consumption, eating-out frequency, breakfast frequency, household income, education level, and marital status.

## 4. Discussion

This study retrospectively compared changes in the food habits and dietary patterns of adults aged ≥65 years between the pre– and during–COVID-19 pandemic periods us-ing 2018–2021 KNHANES data.

The dietary behavior and dietary pattern results demonstrated that the frequency of eating breakfast decreased during COVID-19 among participants who consumed break-fast three or more times a week. While no significant differences in daily meal patterns occurred, the rate of the three–meal–a–day pattern (i.e., “B + L + D”) decreased by approximately 2.49% from “Before COVID-19” (81.92%) to “During COVID-19” (84.41%). In contrast, the frequency of the two–meal–a–day patterns (e.g., “B + L,” “B + D,” and “L + D”) in-creased, indicating a transition toward skipping one meal per day. The decrease in the proportion of people eating three meals a day and concurrent increase in that of people eating two meals a day suggests a rise in the proportion of people skipping meals “During COVID-19” compared with that “Before COVID-19.” This finding is consistent with that of previous studies that reported an increase in the proportion of South Koreans skipping breakfast and lunch during the COVID-19 pandemic [46]. The proportion of meals pre-pared at “home only” and “H+C” increased “During COVID-19” compared with that “Before COVID-19,” reflecting an increase in home–cooked and commercial meals (including delivery meals) and a decrease in institutional meals. In other words, all dietary patterns, including those involving non–commercial food services, decreased during the COVID-19 period.

Overseas studies have reported that the COVID-19 pandemic restricted daily activities, culminating in lifestyle changes, such as weight gain, decreased physical activity, and increased home and delivered meals owing to more time spent at home rather than in active places [12]. Another study analyzed the decline in institutional foodservice for older adults after the COVID-19 outbreak, attributing it to factors such as bans on social gatherings, the suspension of institutional foodservice owing to teleworking, restrictions on outside activities, and the suspension of senior and social–welfare centers, and reported reciprocal effects [26]. The ban on social gatherings, working from home, and restrictions on daily outdoor activities might have elicited an increase in commercial and home meals, excluding institutional foodservices, during the COVID-19 pandemic.

In terms of food insecurity, the number of respondents reporting that they had enough to eat increased between the “Before COVID-19” and “During COVID-19” periods, whereas that of those reporting that they were food insecure decreased. Previous studies on food security have revealed a positive improvement in food insecurity “During COVID-19” compared with that “Before COVID-19,” exhibiting consistency with the results of this study. Food insecurity was reportedly influenced by increased public health awareness of food security and its importance [47]. Another study found that financial and food assistance programs during the pandemic improved access to food and contributed to healthier food intake, depending on the timing and availability of the assistance programs [48]. Considering these findings, nutrition interventions, such as food vouchers and nutrition education, likely contribute to food security.

The intake of fresh fruit and non–starchy vegetables was found to be approximately 50 g lower than the WHO– and WCRF–recommended 400 g both “Before COVID-19” (350.37 g/day) and “During COVID-19” (356.70 g/day) [40,41]. Previous studies have demonstrated that fruit and vegetable consumption in older adults is an indispensable component of a healthy diet and is associated with a range of positive health outcomes and reduced risk of chronic diseases, such as cardiovascular disease, stroke, and cancer [49,50,51]. High fruit and vegetable consumption has also been reported to be beneficial for the immune system [52]. To improve dietary habits, dietary guidelines and nutrition education programs aimed at increasing fruit and vegetable consumption should be developed.

Research on dietary changes has generated inconsistent results, with some studies reporting no significant changes between the pre– and during–pandemic periods [53] and others indicating increased Mediterranean diet intake [53] and elevated fruit and vegetable consumption [54] during COVID-19. These factors are strongly influenced by socio demographic factors, with low–income groups being particularly negatively affected [54,55].

The decrease in energy, carbohydrate, iron, and thiamine intake along with the reduced energy contribution of carbohydrates “During COVID-19” compared with that “Before COVID-19” is potentially related to the decreased intake of cereals and grains between the two periods. However, in this study, the energy contribution of carbohydrates was found to exceed the adequate percentage by 3.79–6.02% in both the “Before COVID-19” (71.02%) and “During COVID-19” (68.79%) groups. Several studies have also reported the energy contribution of carbohydrates in Korean older adults to approximate 70% [32,43]. According to the Korean Dietary Reference Intakes (KDRIs), the Acceptable Macronutrient Distribution Range (AMDR) for carbohydrates is 55–65% (based on the 2020 KDRIs). Previous studies have reported an increased risk of health problems when the energy intake ratio of macronutrients, such as carbohydrates, falls outside the recommended range [56].

No statistically significant difference in sodium intake was noted. However, its intake increased “During COVID-19” (2885.60 mg) compared with that “Before COVID-19” (2816.22 mg) and exceeded the level that mitigates the risk of chronic disease (based on the 2020 KDRIs, 65–74 years: 2100 mg; 75+ years: 1700 mg) by approximately 700–1100 mg [56]. Excessive sodium intake is associated with an increased risk of hypertension and chronic disease; therefore, caution is advised [57,58,59].

Finally, another study found the intake of calcium, vitamin A, and vitamin C to be lower than that recommended by the KDRIs both before and during COVID-19 [56]. A study on calcium intake trends over 20 years among adults aged ≥20 years in South Korea reported that South Koreans generally have a low calcium intake, highlighting the urgent need to increase the availability of calcium–rich foods and implement targeted interventions to increase calcium intake among those most affected by calcium insufficiency, specifically older Koreans [60]. This is because in people aged ≥65 years, these nutrients are important for health, including bone health, connective tissue formation, and antioxidant activity [43]. Therefore, a deficiency of these nutrients potentially leads to health deterioration in older adults; hence, policies that increase the intake of these nutrients are urgently required. In addition, the development of a dietary education program that ensures proper nutrient intake in older Koreans is also necessary.

This study has certain limitations. First, it was a KNHANES–based, cross–sectional study, and like previous studies, it did not allow for the investigation of changes in individual energy, nutrient, and food group intakes, an initiative that would have been possible in a long–term cohort study [30,61]. Second, the KNHANES dietary survey was conducted based on the recall of meals eaten the day before the survey, carrying potential ac-curacy limitations in participants’ recollection of their daily intake [61]. Despite these limitations, this study presents potentially meaningful findings because the KNHANES yields representative dietary data for South Korea and utilizes a representative sample.

## 5. Conclusions

This study analyzed the effects of the COVID-19 pandemic on the dietary habits and nutritional status of people aged ≥65 years in South Korea. According to its findings, the dietary habits and nutritional status of the study group evolved during the pandemic. In particular, the frequency of drinking four or more times a week decreased, and the intakes of certain food groups and nutrients altered.

These changes are presumably related to social distancing and pandemic-induced modifications of daily lifestyles. In addition, certain nutrients, especially vitamin A, vitamin C, and calcium, were found to be below recommended values, and improvements are warranted. Based on these research findings, a systematic approach to improving the nutritional status of older people is imperative. In particular, developing dietary and nutrition education programs that enhance immunity and prevent chronic disease and osteoporosis as well as practical dietary guidelines that increase the intake of deficient nutrients is mandatory.

In addition, this study’s findings provide an important basis for the formulation of nutrition management strategies for older adults in the event of a similar public health crisis in the future. For example, these analyses support programs such as the mobilization of agricultural product bundles for nutritionally vulnerable older adults, the distribution of nutrition kits, and the provision of remote nutrition-counseling services. The continuous monitoring and analysis of changes in the dietary habits of older adults remains necessary post-pandemic.

## Figures and Tables

**Table 1:** Comparison of participants’ general characteristics before and during COVID-19.

Variables	Before COVID-19 (*n* = 2943)		During COVID-19 (*n* = 2916)		Total (*n* = 5859)		*p*-Value ^(1)^
	*n*	%	*n*	%	*n*	%	
Total	2943	47.07	2916	52.93	5859	100.00	-
Sex							0.331
Male	1234	44.76	1252	46.01	2486	45.43	
Female	1709	55.24	1664	53.99	3373	54.57	
Age (years)							0.919
65–74	1688	58.98	1672	59.16	3360	59.07	
≥75	1255	41.02	1244	40.84	2499	40.93	
Marital status							0.878
Married	2919	99.17	2890	99.21	5809	99.19	
Single	24	0.83	26	0.79	50	0.81	
Education level							0.605
High school graduate or lower	1919	66.12	1685	64.16	3604	65.12	
High school diploma	513	20.95	524	22.45	1037	21.72	
College degree or higher	309	12.93	315	13.39	624	13.17	
Region							0.317
City	2132	78.57	1995	74.19	4127	76.26	
Rural area	811	21.43	921	25.81	1732	23.74	
Employment status							0.003
Employed	934	33.20	1002	38.86	1936	36.09	
Unemployed	1810	66.80	1525	61.14	3335	63.91	
Household income							0.557
Low	1411	44.87	1323	42.23	2734	43.48	
Middle-low	812	28.15	837	29.62	1649	28.93	
Middle-high	445	16.86	450	16.87	895	16.87	
High	266	10.12	280	11.27	546	10.73	

^(1)^* p*-value according to the chi-square test.

**Table 2:** Comparison of health-related behaviors before and during COVID-19.

Variables	Before COVID-19 (*n* = 2943)		During COVID-19 (*n* = 2916)		Total (*n* = 5859)		*p*-Value ^(1)^
	*n*	%	*n*	%	*n*	%	
Smoking status							
Current smoker	249	9.22	267	9.73	516	9.49	0.675
Past smoker	825	29.65	796	30.47	1621	30.08	
Non-smoker	1830	61.13	1795	59.80	3625	60.43	
Drinking frequency							
<1 time/month	1910	63.91	2000	68.69	3910	66.43	0.006
1–4 times/month	527	19.19	465	16.48	992	17.76	
2–3 times/week	252	9.03	233	8.89	485	8.96	
≥4 times/week	219	7.86	163	5.94	382	6.85	
Stress level							
Severe stress	106	3.21	96	3.17	202	3.19	0.287
Moderate stress	407	13.92	388	13.18	795	13.53	
Mild stress	1478	52.35	1538	55.37	3016	53.94	
No stress	913	30.52	830	28.29	1743	29.34	
Exercise							
<1 day/week	2244	80.18	1977	76.91	4221	78.51	0.057
1–2 days/week	88	3.58	100	3.99	188	3.79	
3–4 days/week	134	5.34	136	5.41	270	5.37	
≥5 days/week	282	10.90	315	13.69	597	12.33	
Obesity status							
Underweight (BMI < 18.5)	77	2.46	91	3.15	168	2.82	0.164
Normal (18.5 ≤ BMI < 23)	1000	35.20	940	33.83	1940	34.49	
Overweight (23 ≤ BMI < 25)	765	26.78	712	25.09	1477	25.90	
Obesity (BMI ≥ 25)	1055	35.56	1054	37.93	2109	36.80	
Chewing ability							
Very inconvenient	288	8.41	276	9.39	564	8.92	0.063
Inconvenient	827	27.92	698	24.20	1525	25.96	
Normal	533	18.19	510	18.69	1043	18.45	
Not inconvenient	529	18.98	603	21.22	1132	20.16	
Not inconvenient at all	725	26.50	770	26.51	1495	26.51	

^(1)^* p*-value according to the chi-square test.

**Table 3:** Comparison of dietary behaviors before and during COVID-19.

Variables	Before COVID-19 (*n* = 2943)		During COVID-19 (*n* = 2916)		Total (*n* = 5859)		*p*–Value ^(1)^
	*n*	%	*n*	%	*n*	%	
**Breakfast**							<0.001
5–7 times/week	2741	93.00	2693	91.56	5434	92.24	
3–4 times/week	85	3.21	61	2.18	146	2.66	
1–2 times/week	40	1.27	66	2.55	106	1.95	
Rarely (<1 time/week)	77	2.52	96	3.72	173	3.15	
**Snack**							0.6492
Yes	2672	91.27	2641	90.87	5313	91.06	
No	271	8.73	275	9.13	546	8.94	
**Daily meal pattern** ^(2)^							0.2935
B + L	108	3.53	107	3.81	215	3.68	
B + D	201	6.99	228	8.24	429	7.65	
L + D	120	4.21	144	5.28	264	4.78	
B + L + D	2485	84.41	2412	81.92	4897	83.09	
Others	26	0.86	23	0.74	49	0.80	
**Home**							0.6488
Eating	2823	95.82	2815	96.10	5638	95.97	
Not eating	120	4.18	101	3.90	221	4.03	
**Commercial location**							0.0715
Eating	1276	45.60	1192	42.34	2468	43.88	
Not eating	1667	54.40	1724	57.66	3391	56.12	
**Institutional location**							<0.001
Eating	209	7.01	94	3.28	303	5.04	
Not eating	2734	92.99	2822	96.72	5859	94.96	
**Frequency of eating** **–out**							<0.001
≥1 time/day	130	5.13	97	3.91	227	4.49	
5–6 times/week	203	7.13	155	5.49	358	6.26	
3–4 times/week	249	8.97	180	6.21	429	7.51	
1–2 times/week	819	28.33	515	18.91	1334	23.34	
1–3 times/month	938	31.54	1027	34.46	1965	33.08	
Rarely	604	18.90	942	31.03	1546	25.32	
**Food security**							0.0056
Sufficiently food secure	1395	48.57	1585	54.88	2980	51.91	
Mildly food insecure	1377	46.29	1180	40.18	2557	43.06	
Moderately/Severe food insecure	168	5.14	150	4.94	318	5.03	

^(1)^* p*-value according to the chi-square test; ^(2)^ B: breakfast, L: lunch, D: dinner, other: ≤1 meal.

**Table 4:** Daily food group intake before vs. during COVID-19.

Food Group	Before COVID-19 (*n* = 2943)	During COVID-19 (*n* = 2916)	Total (*n* = 5859)	Unadjusted *p*-Value ^(1)^	Adjusted *p*-Value ^(2)^
Total food (g)	1243.00 ± 16.14 ^(3)^	1255.33 ± 19.24	1249.53 ± 12.47	0.2805	0.0065
Cereals and grains (g)	260.13 ± 2.74	241.30 ± 2.77	250.16 ± 1.91	<0.0001	0.0002
Potatoes and starches (g)	33.62 ± 2.25	41.24 ± 2.33	37.65 ± 1.62	0.0539	0.0137
Sugars and sweets (g)	5.85 ± 0.30	5.52 ± 0.26	5.68 ± 0.20	0.6031	0.7364
Legumes (g)	43.90 ± 1.99	51.31 ± 2.08	47.82 ± 1.44	0.0070	0.0023
Seeds and nuts (g)	7.96 ± 0.70	8.04 ± 0.70	8.00 ± 0.49	0.9801	0.8785
Vegetables ^(4)^ (g)	304.28 ± 5.08	327.82 ± 5.67	316.74 ± 3.85	0.0005	<0.0001
Mushrooms (g)	4.64 ± 0.56	4.45 ± 0.48	4.54 ± 0.37	0.9127	0.8652
Fruits ^(5)^ (g)	166.20 ± 5.99	160.91 ± 6.15	163.40 ± 4.28	0.9100	0.7176
Seaweed (g)	29.63 ± 2.33	32.36 ± 2.60	31.08 ± 1.74	0.2324	0.2009
Meat and poultry (g)	60.64 ± 2.72	61.60 ± 2.57	61.15 ± 1.86	0.9837	0.7465
Eggs (g)	22.18 ± 0.94	25.42 ± 0.99	23.90 ± 0.67	0.0149	0.0091
Fishes and shell fishes (g)	100.62 ± 4.07	90.60 ± 3.86	95.32 ± 2.75	0.1433	0.1565
Milks and dairy products (g)	63.72 ± 3.03	73.11 ± 3.40	68.69 ± 2.29	0.0206	0.0060
Oils and fats (g)	3.82 ± 0.14	4.15 ± 0.17	4.00 ± 0.11	0.0267	0.0043
Beverages (g)	59.48 ± 3.60	59.92 ± 3.76	59.71 ± 2.59	0.9569	0.9265
Seasonings (g)	26.60 ± 0.61	26.73 ± 0.77	26.67 ± 0.50	0.9501	0.6483
Other food (g)	0.91 ± 0.43	0.74 ± 0.15	0.82 ± 0.22	0.6863	0.6749

^(1)^* p*-value according to the *t*-test; ^(2)^ adjusted for sex, age, and energy intake; ^(3)^ mean ± standard error; ^(4)^ including salted vegetables, kimchi, and vegetable juice; ^(5)^ including fruits preserved in sugar, jam, and fruit juice.

**Table 5:** Plant food intake before vs. during COVID-19.

Plant Food (g/day)	Before COVID-19 (*n* = 2943)	During COVID-19 (*n* = 2916)	Total (*n* = 5859)	Unadjusted *p*-Value ^(1)^	Adjusted *p*-Value ^(2),(3)^
Fresh fruits ^(5)^	165.46 ± 5.95 ^(4)^	157.33 ± 6.10	161.16 ± 4.25	0.4041	0.6372
Non-starchy vegetables ^(6)^	184.91 ± 4.43	199.37 ± 4.81	192.56 ± 3.26	0.0233	0.0040
Fresh fruits and non-starchy vegetables	350.37 ± 8.42	356.70 ± 8.64	353.72 ± 5.95	0.5279	0.2004

^(1)^* p*-value according to the *t*-test; ^(2)^* p*-value according to the generalized linear model (GLM); ^(3)^ adjusted for sex, age, and energy intake; ^(4)^ mean ± standard error; ^(5)^ excluding fruits preserved in sugar, jam, and fruit juice; ^(6)^ including salted vegetables, kimchi, and vegetable juice.

**Table 6:** Nutrient intake before vs. during COVID-19.

Nutrients	Before COVID-19 (*n* = 2943)	During COVID-19 (*n* = 2916)	Total (*n* = 5859)	Unadjusted *p*-Value ^(1)^	Adjusted *p*-Value ^(2),(3)^
Energy (kcal)	1617.70 ± 14.24 ^(4)^	1579.98 ± 16.77	1597.73 ± 11.08	<0.0001	0.0497
Carbohydrate (g)	272.43 ± 2.34	258.98 ± 2.44	265.31 ± 1.67	0.0015	0.0003
Protein (g)	55.51 ± 0.67	56.58 ± 0.82	56.08 ± 0.53	0.1332	0.0001
Fat (g)	28.60 ± 0.52	31.54 ± 0.68	30.15 ± 0.43	0.0003	<0.0001
Fiber (g)	25.77 ± 0.37	27.27 ± 0.39	26.56 ± 0.27	0.0009	<0.0001
Calcium (mg)	457.98 ± 8.13	471.91 ± 7.93	465.35 ± 5.66	0.0906	0.0067
Phosphorus (mg)	911.70 ± 10.61	938.87 ± 12.39	926.08 ± 8.16	0.0192	<0.0001
Iron (mg)	9.79 ± 0.16	8.54 ± 0.16	9.13 ± 0.11	<0.0001	<0.0001
Sodium (mg)	2816.22 ± 38.77	2885.60 ± 45.64	2852.95 ± 30.48	0.1247	0.6253
Magnesium (mg)	300.07 ± 3.52	301.57 ± 3.96	300.87 ± 2.66	0.3785	0.0117
Potassium (mg)	2524.38 ± 34.25	2599.63 ± 36.18	2564.22 ± 24.85	0.0457	<0.0001
Zinc (mg)	9.19 ± 0.10	9.25 ± 0.12	9.22 ± 0.08	0.3771	0.0059
Vitamin A (μg RAE)	323.49 ± 12.08	342.88 ± 10.00	333.75 ± 7.68	0.2692	0.1063
Carotene (μg)	2725.37 ± 82.73	2874.32 ± 86.64	2804.22 ± 59.59	0.2205	0.0967
Retinol (μg)	96.40 ± 9.27	103.34 ± 5.61	100.08 ± 5.22	0.6583	0.4892
Thiamine (mg)	1.06 ± 0.01	0.95 ± 0.01	1.00 ± 0.01	<0.0001	<0.0001
Riboflavin (mg)	1.18 ± 0.02	1.22 ± 0.02	1.20 ± 0.01	0.0408	0.0004
Niacin (mg)	9.99 ± 0.14	9.72 ± 0.16	9.85 ± 0.10	0.6609	0.8147
Vitamin D (μg)	2.72 ± 0.17	2.58 ± 0.11	2.65 ± 0.10	0.7035	0.8619
Vitamin E (mg α-TE)	5.33 ± 0.09	5.78 ± 0.09	5.57 ± 0.07	0.0001	<0.0001
Vitamin C (mg)	55.76 ± 1.73	62.71 ± 1.83	59.44 ± 1.25	0.0083	0.0010
Folic acid (μg DFE)	309.50 ± 4.21	320.65 ± 4.71	315.41 ± 3.17	0.0315	0.0002
Energy distribution (%)					
Carbohydrate	71.02 ± 0.26	68.79 ± 0.30	69.84 ± 0.19	<0.0001	<0.0001
Protein	13.62 ± 0.09	14.10 ± 0.10	13.87 ± 0.07	<0.0001	0.0003
Fat	15.36 ± 0.20	17.11 ± 0.23	16.29 ± 0.15	<0.0001	<0.0001

^(1)^* p*-value according to *t*-test; ^(2)^* p*-value according to the generalized linear model (GLM); ^(3)^ adjusted for sex, age, and energy intake; ^(4)^ mean ± standard error.

**Table 7:** Associations of the pre- and during-COVID-19 pandemic periods with nutritional insufficiency.

	Total ^(1)^		Energy		Calcium		Iron		Vitamin A		Riboflavin	
	*n*	%	*n*	%	*n*	%	*n*	%	*n*	%	*n*	%
Before COVID-19	503	16.45	947	31.57	2312	77.72	859	28.10	2383	81.00	1393	46.67
During COVID-19	619	20.76	1037	35.59	2195	73.82	1164	39.34	2263	77.12	1299	43.24
*p*-value ^(2)^	0.0012		0.014		0.0184		<0.0001		0.0103		0.0822	
Model 1	1.350 (1.121–1.626) ** ^(3)^		1.198 (1.026–1.399) *		0.781 (0.652–0.936) **		1.652 (1.398–1.952) ***		0.809 (0.675–0.970) **		0.859 (0.727–1.014)	
Model 2	1.375 (1.142–1.655) ***		1.221 (1.046–1.426) *		0.796 (0.663–0.956) *		1.676 (1.420–1.978) ***		0.821 (0.686–0.984) *		0.875 (0.742–1.031)	
Model 3	1.314 (1.092–1.580) **		1.171 (1.003–1.360) *		0.797 (0.663–0.959) *		1.632 (1.384–1.926) ***		0.818 (0.683–0.978) *		0.851 (0.721–1.003)	
Model 4	1.419 (1.170–1.721) ***		1.172 (0.998–1.376)		0.783 (0.647–0.947) *		1.697 (1.429–2.016) ***		0.834 (0.692–1.005)		0.841 (0.712–0.995) *	

^(1)^ Dependent variables were calculated based on the percentage of participants who consumed less than 75% of the estimated energy requirement (EER) for energy and less than the estimated adequate requirement (EAR) for vitamin A, riboflavin, calcium, and iron (0: nutritional sufficiency, and 1: nutritional insufficiency); ^(2)^* p*-value according to the chi-square test; ^(3)^ odds ratio (95% confidence interval); independent variable: before/during COVID-19 (0: before COVID-19, 1: during COVID-19); Model 1: unadjusted; Model 2: adjusted for sex and age; Model 3: adjusted for sex, age, smoking status, drinking status, and stress and weight status; Model 4: adjusted for sex, age, smoking status, drinking status, stress and weight status, food security, snack consumption, eating-out frequency, breakfast frequency, education level, household income, and marital status; * *p* < 0.05, ** *p* < 0.01, and *** *p* < 0.001.

## Data Availability

All data were obtained from the Korea Disease Control and Prevention Agency and are available with the permission of the Korea Disease Control and Prevention Agency. The data in this study were from the Korea National Health and Nutrition Examination Survey.
